# Review of M. Bernard’s Theory of an Hepatico-Renal Circulation

**Published:** 1853-01

**Authors:** D. B. Phillips

**Affiliations:** Assistant Surgeon, U. S. N., and Member of the Academy of Natural Sciences of Philadelphia


From the Medical Examiner.

*Review of M. Bernard's Theory of an Hepatico-Renal Circulation. by* D. B. PHILLIPS, M. D., Assistant Surgeon, U. S. N., and Member of the Academy of Natural Sciences of Philadelphia.

In reviewing the recent theories upon physiology, I have met with nothing so well calculated to arrest the attention, as the assertion of M. Bernard regarding the functions of the inferior vena cava, renal, portal, and azygos veins, during the process of digestion.

His views may be briefly stated as follows: Whilst the digestive process is going on, the portal veins imbibe the fluid contents (or a sufficient portion of them) from the stomach, and thus become engorged; the liver not being able to receive the whole of this, and still carry on its healthy office, a portion of this blood is sent into the inferior cava by a direct communication between it and the portal vein; this blood, charged with the materials absorbed from the stomach, passes up the vena cava until within a short distance of the diaphragm, here the coats of the inferior cava contract upon its contents, forcing the blood down again as far as the renal veins, but owing to the existence of valves just below the mouths of these veins, it cannot pass lower down, but is forced to enter those veins and thus pass on to the kidneys, where any poisonous properties are eliminated, without having entered into the general circulation. The blood from the lower extremities, he thinks, is returned to the heart by means of the two azygos veins, which, he affirms, have their origin below the valves before alluded to. It is with very great diffidence that I attempt an investigation, or presume to question such high authority as M. Bernard's, yet it seems to me that there are some insuperable objections to his theory when applied to the human subject. In the first place, after having witnessed careful dissections made for this purpose, I have never been able to discover any trace of the communication between the inferior cava and the portal vein, other than through the hepatic veins. Secondly, anatomists tell us that there are no valves in the inferior cava: this is positively affirmed by Mr. Wilson. Thirdly, the same author tells us, that the azygos veins frequently anastomose with the renal veins; in which case it would be evident that the existence of valves in the inferior cava would be no hindrance to the passage of the blood of the portal veins into the general circulation. These reasons alone seem to me sufficient to refute M. Bernard's theory; but apart from these, there still exist others of even more stubborn nature. Suppose we admit the existence of communication, valves, and proper arrangement of the azygos veins, as stated by M. Bernard. Then, during the whole digestive process, there must of necessity be an antagonism between the renal arteries and veins; the first driving arterial blood into the kidneys, with all the force of a powerful muscle, such as is the heart, and the other acting only by the comparatively feeble contractility of the coats of the cava, it would be self-evident that the latter would be prevented from entering the kidneys from the little resistance it could afford. But even were the antagonism perfect, and did both vessels discharge their contents into the kidneys with equal force, then for the space of fifteen hours out of the twenty-four, the kidneys would become the recipients of an amount of blood, even quintuple the quantity of urine evacuated.

If M. Bernard's theory is correct, there could of course be no blood returned from the kidneys during the whole process of digestion.—Now, adopting five hours as the length of time occupied in this process, and allowing three meals per day; we should have no circulation in the kidneys, (or rather no return of blood from them) for fifteen hours out of the twenty-four. What then becomes of the enormous amount of blood which has been driven into these organs by both arteries and veins during the whole of this period? M. B. has given us no solution to this mystery. Thus, then, to sum up: The communication between the vena cava and portal system cannot be demonstrated; except, perhaps, as an abnormal occurrence in the human subject. The existence of valves in the inferior cava are denied by the best anatomical authority; the existence of anastomoses between the azygos and renal veins, proven to be of frequent occurrence; and, above all, there still exists the objection, that the kidneys receive an amount of blood vastly exceeding the quantity of urine eliminated. I think that with these difficulties unexplained, that we must even hesitate to adopt a theory which, although beautiful in its design and imagination, seems thus far at least to be wanting in facts and reasons to support it.

